# An interpretable analysis of the depressive status and its influencing factors in elderly patients with stroke

**DOI:** 10.3389/fneur.2026.1799200

**Published:** 2026-04-09

**Authors:** Huiting Xu, Pan Xia

**Affiliations:** 1 Intensive Care Unit, Yancheng No.1 People’s Hospital, Affiliated Hospital of Medical School, Nanjing University, Yancheng, Jiangsu, China; 2 Department of Neurology, Nanjing Gaochun People's Hospital, Nanjing, Jiangsu, China

**Keywords:** depression, machine learning, mental health, stroke, XGBoost

## Abstract

**Background:**

This study aims to develop and validate an Gradient Boosting algorithm (XGBoost) model for predicting the risk of depression in elderly stroke patients, and simultaneously identify the key risk factors.

**Methods:**

A cross-sectional survey was conducted on 260 elderly patients with stroke. Depression scales were used for screening, and XGBoost was employed to analyze the data to identify the key influencing factors and rank them according to their predictive importance.

**Results:**

Among the elderly stroke patients surveyed, the prevalence of depression was 24.615%. According to the XGBoost model, the importance of various factors was ranked as follows: sleep status, social participation, marital status, history of falls, and educational level.

**Conclusion:**

Depression in elderly stroke patients should not be overlooked. Clinical medical staff pay more attention to factors such as sleep status, social participation, marital status, history of falls, and educational level. Clinical medical staff should formulate individualized intervention strategies based on the specific conditions of elderly stroke patients to effectively reduce the risk of depression.

## Introduction

With the aging of the population and the rise in unhealthy lifestyles, the incidence of stroke continues to increase, representing a major public health challenge globally. Stroke not only impairs speech and physical function but also significantly affects mental and psychological health, leading to emotional disturbances, anxiety, and depression, which are often more challenging to manage than physical disabilities (1). Approximately one-third of stroke patients experience depressive symptoms at various stages following the event, with cumulative incidence rates within 5 years ranging from 39% to 52% (2). Post-stroke depression manifests with symptoms such as depressed mood, loss of interest, sleep disturbances, and somatic complaints (3). Studies indicate that compared to stroke survivors without depressive symptoms, those with post-stroke depression exhibit more severe deficits in activities of daily living, pronounced cognitive impairments, and greater disability, leading to poorer functional recovery outcomes, higher recurrence rates, and increased mortality (4). Furthermore, post-stroke depression hinders full participation in essential rehabilitation training and significantly prolongs hospitalization, with an average extension of 8.5 days (5, 6). This not only escalates medical resource utilization but also imposes additional economic burdens and psychological stress on patients and their families (6, 7). Furthermore, existing literature indicates that alongside neurological impairment, psychological status is a key determinant of quality of life following stroke (8). While traditional statistical methods, such as regression analysis, have identified risk factors and offered foundational insights, their predictive validity within the post-stroke depression population remains significantly constrained (9, 10). This limitation stems from the complex, multi-factorial etiology of the condition, characterized by substantial heterogeneity and intricate non-linear interactions that conventional models fail to adequately capture (11, 12). Consequently, developing robust predictive tools for early screening and risk identification is essential to facilitate timely interventions, thereby preventing the progression of depression and mitigating its adverse burden. The XGBoost algorithm is an ensemble-based boosting method that constructs weak learners by optimizing a structured loss function. It employs techniques such as pre-sorting and weighted quantiles to enhance computational efficiency and generalization capability while effectively mitigating overfitting (13). SHAP (Shapley Additive exPlanations) values, grounded in cooperative game theory, quantify the magnitude and direction of each feature’s contribution to model predictions, thereby providing an intuitive and interpretable feature importance analysis for machine learning models (14). Within this methodological framework, this study systematically investigates the influencing factors and their interaction patterns for depression in elderly stroke patients by employing an XGBoost model combined with SHAP interpretation. The research aims to offer practical insights for clinical healthcare professionals, facilitating early identification of high-risk groups and promoting the development of targeted prevention and intervention strategies to reduce the incidence and impact of post-stroke depression in the elderly population.

## Methods

### Participants

From May 2024 to June 2025, researchers conducted a survey among elderly stroke patients using convenience sampling at two hospitals in Nanjing and Yancheng. Prior to questionnaire distribution, the research team explained the study purpose, main content, and data confidentiality measures to all potential participants. Questionnaires were administered only after obtaining explicit informed consent from the participants. This study was conducted on an anonymous and voluntary basis. After excluding 15 invalid questionnaires, 260 valid participants remained, corresponding to an effective response rate of 94.5%.

Inclusion criteria: meeting the diagnostic criteria for stroke, with newly diagnosed ischemic or hemorrhagic stroke confirmed by CT and/or MRI; hospitalized stroke patients within 7 to 30 days after onset (when depressive symptoms typically emerge and patients are stable for assessment); age ≥ 60 years (15, 16); clear consciousness; and voluntary participation in this study.

Exclusion criteria: presence of speech or cognitive impairments, intellectual disability, or concurrent participation in other stroke-related studies.

### Measures

#### Demographic characteristics

Gender, Age, Educational level, Residence, Monthly household income(¥), Living arrangement, Marital status, History of cerebrovascular disease, History of falls (≥1 unintentional fall in the year prior to index stroke onset, irrespective of injury outcome), Social participation (frequency of engagement in leisure/recreational activities, family/friend gatherings, and community events), Physical activity, Sleep status (evaluated based on: sleep onset latency, nocturnal sleep duration, daytime alertness, and sedative/hypnotic use), Smoking, Drinking, Number of medications, History of diabetes [met the diagnostic criteria for diabetes mellitus (17)], History of hypertension [met the diagnostic criteria for hypertension (18)].

#### The 15-item geriatric depression scale (GDS-15)

GDS is a well-established instrument for assessing depression levels in older adults. The 15-item short form (GDS-15), developed by Sheikh et al. (19) as a simplified version of the original 30-item scale, retains strong psychometric validity while improving clinical feasibility. Each item adopts a simple yes/no response format (scored as 1 or 0, respectively), yielding a total score ranging from 0 to 15. Scores are interpreted as follows: 0–4 indicates no depression, 5–9 suggests mild depression, and ≥10 indicates moderate to severe depression. The GDS-15 has been extensively validated in elderly and post-stroke populations, demonstrating high internal consistency, good test–retest reliability, and favorable sensitivity/specificity for detecting major depressive disorder (20–25). Its concise format and minimal somatic item content contribute to strong tolerability among elderly stroke patients, making it a practical and reliable screening tool in this population. In the present study, the GDS-15 exhibited excellent internal consistency, with a Cronbach’s *α* of 0.891.

### Statistical analysis

Although machine learning algorithms such as XGBoost have the capability to automatically learn complex patterns from data, to further enhance the clinical interpretability of the model and ensure that the variables ultimately included are known to be associated with the clinical outcome, we first employed univariate analysis and binary logistic regression to preliminarily screen for meaningful variables (*p* < 0.05). Additionally, this preprocessing step helps reduce the dimensionality of the features, thereby saving computational resources to some extent and improving the efficiency of subsequent modeling. Statistical analysis was performed using SPSS 27.0. Categorical variables were summarized as frequencies and percentages, with between-group comparisons conducted using the *χ*^2^ test; statistical significance was defined as *p* < 0.05. Predictive modeling was implemented in Python 3.9 using the Pandas, NumPy, and XGBoost libraries. The analysis dataset comprised 260 complete records involving 17 integer (int64) variables with no missing data. Using predictors identified through univariate screening, an XGBoost model was trained to classify depression. This ensemble method integrates sequentially constructed decision trees, with key hyperparameters including maximum tree depth (max_depth), number of trees (n_estimators), and learning rate (default 0.1); other parameters were maintained at their default settings. Hyperparameter tuning was performed via GridSearchCV with 5-fold cross-validation to balance predictive performance and computational cost. The final model was selected based on the highest cross-validated accuracy. SHAP is a method designed for tree-based models. We used the shap Python package (version 0.46.0) and applied explainer = shap. Tree Explainer (model_xgb) to compute SHAP values.

## Results

### General characteristics of the participants

Depressive symptoms were present in 24.615% of the participants. The baseline characteristics of the depression and non-depression groups are summarized in Table 1.

**Table 1:** General characteristics of the participants.

Variables	Total	Depression		*χ* ^2^	*p*
		No	Yes		
Gender				2.055	0.152
Male	138	109	29		
Female	122	87	35		
Age (Years)				0.229	0.892
60–70	124	92	32		
71–80	113	86	27		
>80	23	18	5		
Educational level					
Primary and Junior High School	181	128	53	7.436	0.024
High School	45	40	5		
University and Above	34	28	6		
Residence				1.809	0.179
Urban	186	136	50		
Rural	74	60	14		
Monthly household income(¥)				2.511	0.285
<3,000	86	62	24		
3,001–6,000	88	64	24		
>6,000	86	70	16		
Living arrangement					
Live with family	221	173	48	6.659	0.010
Live alone	39	23	16		
Marital status					
Married	201	161	40	10.611	0.001
Unmarried/divorced/widowed	59	35	24		
History of cerebrovascular disease				0.348	0.555
No	175	130	45		
Yes	85	66	19		
History of falls				13.927	<0.001
No	199	161	38		
Yes	61	35	26		
Social participation				16.427	<0.001
Frequently	76	68	8		
Sometimes	114	73	41		
Never	70	55	15		
Physical activity				1.632	0.442
Daily	63	44	19		
Sometimes	116	88	28		
Never	81	64	17		
Sleep status				27.116	<0.001
Good / Very Good	104	87	17		
Average	93	77	16		
Poor / Very Poor	63	32	31		
Smoking				0.029	0.864
No	185	140	45		
Yes	75	56	19		
Drinking				1.091	0.296
No	212	157	55		
Yes	48	39	9		
Number of medications				5.534	0.063
<2 types	148	116	32		
3–4 types	65	42	23		
>5 types	47	38	9		
History of diabetes				19.443	<0.001
No	176	147	29		
Yes	84	49	35		
History of hypertension				7.466	0.006
No	140	115	25		
Yes	120	81	39		

### Multivariate binary logistic regression analysis

A logistic regression analysis was performed with depressive status among older adults with stroke as the dependent variable, incorporating educational level, living arrangement, marital status, history of falls, social participation, sleep status, history of diabetes, and history of hypertension as independent variables. Multivariate binary logistic regression analysis revealed that a high school education level served as a protective factor against depression in elderly stroke patients. In contrast, unmarried/divorced/widowed status, history of falls, absence of social participation, and poor/very poor sleep status were identified as risk factors for depression in this population, as shown in Table 2.

**Table 2:** Multivariate binary logistic regression analysis of depressive status in older adults with stroke.

Risk factor	Reference factor	B	SE	Waldx^2^	p	OR	95% CI
Educational level	Primary and Junior High School						
High School		−1.244	0.570	4.755	0.029	0.288	0.094–0.882
University and Above		0.310	0.637	0.237	0.626	1.364	0.391–4.754
Living arrangement	Live with family						
Live alone		0.293	0.550	0.283	0.595	1.340	0.456–3.940
Marital status	Married						
Unmarried/divorced/widowed		1.519	0.476	10.204	0.001	4.567	1.798–11.598
History of falls	No						
Yes		1.346	0.422	10.167	0.001	3.840	1.679–8.781
Social participation	Frequently						
Sometimes		1.504	0.504	8.917	0.003	4.502	1.677–12.084
Never		1.533	0.591	6.729	0.009	4.632	1.455–14.749
Sleep status	Good / Very Good						
Average		−0.266	0.464	0.328	0.567	0.767	0.309–1.903
Poor / Very Poor		1.674	0.456	13.477	<0.001	5.331	2.182–13.027
History of diabetes	No	0.764	0.420	3.304	0.069	2.146	0.942–4.890
History of hypertension	No	0.754	0.433	3.036	0.081	2.125	0.910–4.961

### XGBoost model results

#### Optimal feature subset

**Figure 1:**
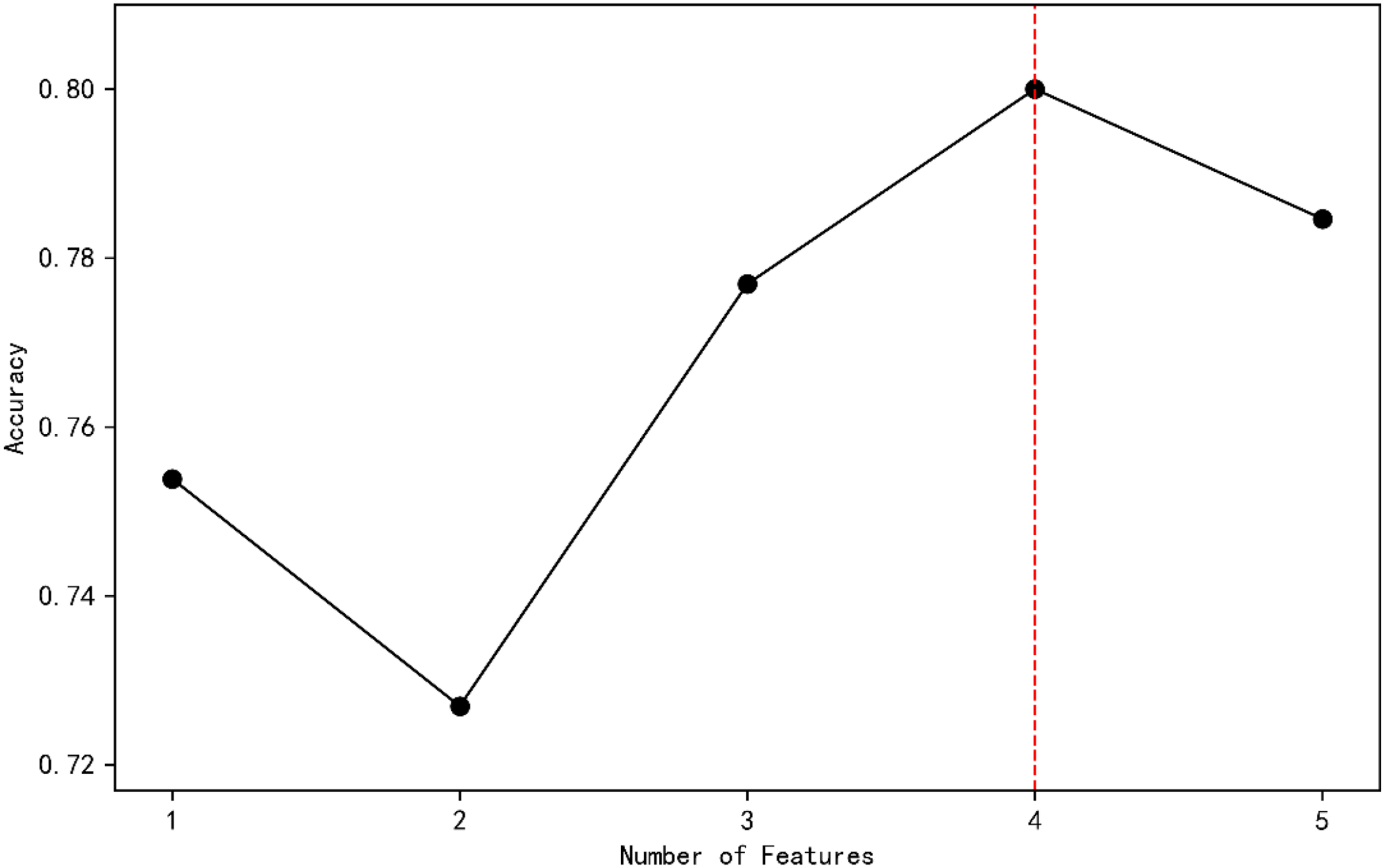
Line graph of the optimal feature subset screening.

Independent variables showing statistical significance (*p* < 0.05) in binary logistic regression analysis were incorporated into the XGBoost model, with post-stroke depression as the dependent variable. To evaluate model performance and determine the optimal feature subset, 5-fold cross-validation was employed to assess classification accuracy across different numbers of retained features. Accuracy values range from 0 to 1, with higher scores indicating superior predictive performance. The results indicated that the highest cross-validation accuracy (0.800) was achieved when the feature subset size was 4 (Figure 1).

#### Feature importance ranking

**Figure 2:**
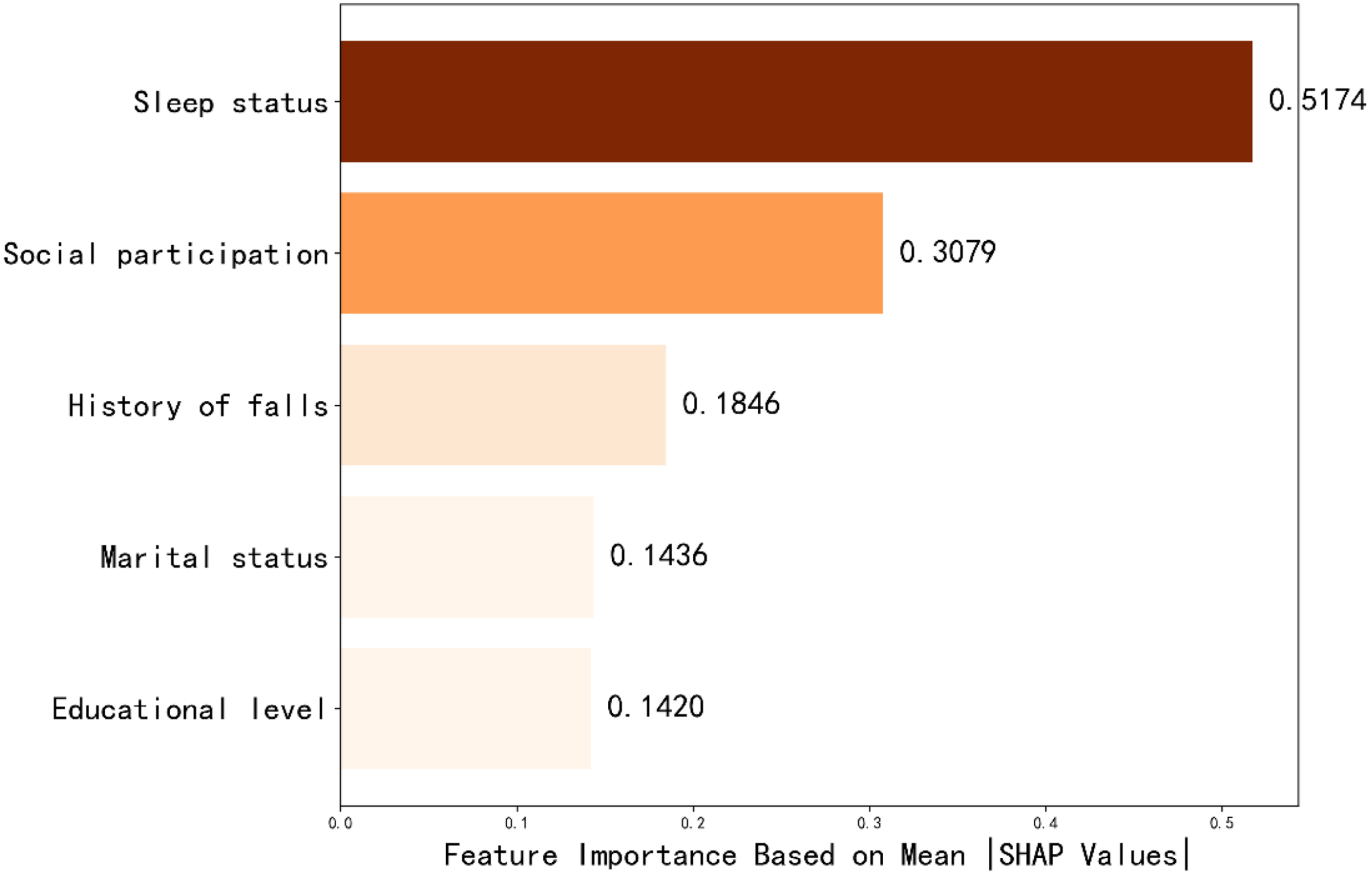
Feature importance ranking.

**Figure 3:**
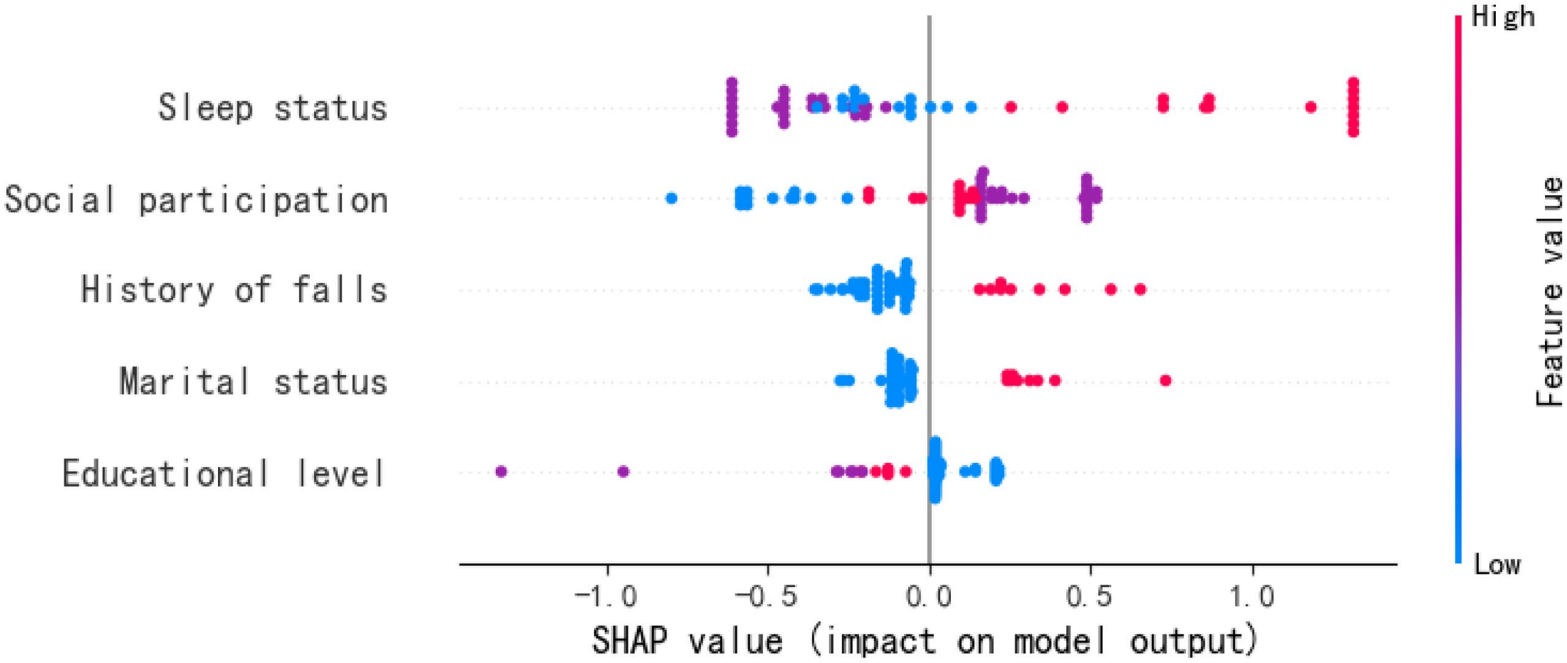
SHAP value (impact on model output).

Based on the optimal feature subset, the dataset was divided into training and test sets in an 8:2 ratio to construct an optimized XGBoost classification model. The identified optimal hyperparameters were max_depth = 4 and n_estimators = 27. SHAP analysis was employed to elucidate the contribution of each feature to the prediction of post-stroke depression in elderly patients. The SHAP feature importance bar plot (Figure 2) displays the following features in descending order of mean absolute SHAP value: Sleep status, Social participation, Marital status, History of falls, and Educational level. The SHAP density scatter plot (Figure 3) further illustrates the relationship between feature value distribution and predictive outcomes.

## Discussion

Our study found that the prevalence of depression among elderly stroke patients was 24.615%, which is lower than the range reported in previous studies (26–28). This discrepancy may be attributed to differences in the survey regions and inclusion criteria.

Depression in elderly stroke patients can manifest as low mood, sleep disturbances, cognitive impairment, and in severe cases, may lead to suicidal behavior (29, 30). Therefore, it is essential to identify the influencing factors of depression in this population and implement early scientific prevention and treatment to reduce associated risks (31). To clarify the relevant influencing factors and develop targeted interventions, this study further employed the XGBoost algorithm to identify and rank the risk factors for depression in elderly stroke patients. The results showed that, in descending order of influence, the key factors are: sleep status, social participation, marital status, history of falls, and educational level.

Our study found that sleep status is a factor influencing depression in elderly stroke patients, consistent with previous research (32). Furthermore, our study further revealed that sleep status ranked as the most significant influencing factor for depression in this population. Poorer sleep quality was associated with a higher probability of depression following stroke. The physiological mechanisms underlying the relationship between sleep and depression remain incompletely understood; however, existing studies suggest that multiple pathways may be involved. Sleep bidirectionally regulates immune responses, thereby affecting the risk of depression (32). Sleep disorders can increase pro-inflammatory factors such as IL-6 and CRP, which may promote depression by disrupting neuronal excitability and HPA axis function. Chronic sleep deprivation can disturb monoamine neurotransmitter systems (e.g., serotonin) and HPA axis negative feedback, thereby exacerbating mood dysregulation (33). Additionally, dysfunction of the suprachiasmatic nucleus may lead to abnormal expression of clock genes, potentially impairing brain regions associated with emotional regulation (34). Our findings also suggest that healthcare professionals should actively create a comfortable sleep environment for elderly stroke patients during hospitalization, assist them in establishing healthy sleep habits, avoid stimulant substances, and improve sleep quality to reduce the incidence of depression.

Furthermore, our study identified social participation as a risk factor influencing depression in elderly stroke patients. Physical disability, limited mobility, and declines in psychological and social functioning following stroke often reduce patients’ engagement in social activities. Decreased social participation implies fewer social interactions and less communication with others, which can lead to feelings of loneliness and isolation. The lack of social support and understanding may further increase psychological stress, thereby facilitating the development of depressive symptoms (35). Active social involvement serves as an important pathway for individuals to integrate into society and adapt to social development. It can help alleviate feelings of emptiness, enrich daily life, and enhance life satisfaction, thereby reducing the risk of depression (36). For patients with limited social participation, in addition to active physical rehabilitation, attention should also be given to strengthening social support and encouraging social engagement. Facilitating social integration can improve self-identity and promote positive emotions, which is beneficial for mitigating depressive symptoms and enhancing rehabilitation outcomes. Elderly stroke patients often experience decreased balance and mobility due to physiological aging and functional impairment, which restricts their activity and increases the risk of falls (37). Falls may cause physical trauma or pain in stroke patients, and such injuries can lead to discomfort and suffering, exacerbating the patient’s physical distress. This, in turn, can negatively affect mood and elevate the risk of depressive symptoms. Therefore, when managing stroke patients who have experienced a fall, clinical nursing staff should not only conduct timely physical examinations and provide treatment but also pay attention to the patient’s psychological well-being (38). Offering psychological support and counseling can help patients cope positively with the fall event, reduce fear and anxiety, and enhance self-confidence, thereby preventing the onset of depression. In addition, regular communication with physicians and rehabilitation specialists to jointly develop an effective rehabilitation plan is also an important measure for depression prevention.

Our study found that marital status is a factor influencing depression in elderly stroke patients, with unmarried patients being more susceptible to depression. Most stroke patients suffer from functional impairments, and having a spouse can provide not only adequate care and financial assistance but also enhance their adherence to functional exercise and rehabilitation beliefs through companionship and encouragement (39). Post-stroke patients often experience a strong sense of burden and low mood, and spousal support can offer emotional comfort and strengthen psychological resilience (40), thereby reducing negative emotions. In contrast, patients without a spouse typically receive less family support and face significant life pressures and financial burdens, leading to severe negative emotions. The lack of emotional exchange and a confidant further contributes to poorer mental health, increasing the likelihood of depression. Therefore, healthcare professionals should pay special attention to unmarried patients by assisting them in seeking governmental and social support to alleviate life pressures, offering more care, and reducing their risk of depression.

Our study identified educational level as a factor influencing depression in elderly stroke patients. A higher educational level served as a protective factor against depression in this population. Patients with relatively lower education often had a limited understanding of the disease, lower acceptance of their condition, poorer treatment adherence, and lower awareness of mental health (41), which could lead to excessive worry and fear. In contrast, patients with higher education levels tended to actively acquire disease-related knowledge through multiple channels, possessed a more comprehensive understanding of the disease, demonstrated better treatment compliance, and cooperated more effectively with healthcare professionals during examinations and treatments (42). However, an interesting finding in our study was that education at the university level or above did not show a significant association with depression in elderly stroke patients. This may be attributed to the limited sample size in our survey. We recommend expanding the sample size in future studies to further validate this finding.

### Limitations

Existing models for predicting post-stroke depression vary significantly. Some are based on clinical data, others utilize imaging or genetic modeling, while yet others adopt multimodal approaches (43–45). Several limitations of this study warrant acknowledgment. First, given the cross-sectional design, causal relationships cannot be established; thus, only correlations between identified factors and depression can be inferred. Second, the relatively small sample size recruited from only two hospitals may compromise the external validity and generalizability of the predictive model. Specifically, this study used convenience sampling, and the sample may be biased toward patients with stable conditions and high compliance, which makes it difficult to represent the entire elderly stroke population. Caution should be exercised when generalizing the results to patients with severe illness or cognitive impairment. Third, certain variables, such as sleep status, were assessed using subjective clinical classifications rather than objective measurement tools. Finally, potential confounding factors, including stroke severity (e.g., NIHSS scores) and rehabilitation treatment status, were not accounted for in this analysis. Consequently, we recommend future large-scale multicenter studies employing more objective measures and longitudinal designs to further elucidate the determinants of depression in elderly stroke patients.

## Conclusion

This study employed the XGBoost model to analyze depression-related factors in 260 elderly stroke patients. Ranked in descending order of importance, these influencing factors were: sleep status, social participation, marital status, history of falls, and educational level. The findings suggest that clinical healthcare professionals should regularly screen elderly stroke patients for depression, consider implementing personalized interventions, provide timely psychological counseling, and address modifiable risk factors to reduce the incidence of depression in this population.

## Data Availability

The original contributions presented in the study are included in the article/supplementary material, further inquiries can be directed to the corresponding author.
